# The Clinical Performance of an Office-Based Risk Scoring System for Fatal Cardiovascular Diseases in North-East of Iran

**DOI:** 10.1371/journal.pone.0126779

**Published:** 2015-05-26

**Authors:** Sadaf G. Sepanlou, Reza Malekzadeh, Hossein Poustchi, Maryam Sharafkhah, Saeed Ghodsi, Fatemeh Malekzadeh, Arash Etemadi, Akram Pourshams, Paul D. Pharoah, Christian C. Abnet, Paul Brennan, Paolo Boffetta, Sanford M. Dawsey, Farin Kamangar

**Affiliations:** 1 Digestive Disease Research Institute, Shariati Hospital, Tehran University of Medical Sciences, Tehran, Iran; 2 Division of Cancer Epidemiology and Genetics, National Cancer Institute, Bethesda, Maryland, United States of America; 3 Department of Public Health and Primary Care, University of Cambridge, Cambridge, United Kingdom; 4 International Agency for Research on Cancer, Lyon, France; 5 The Tisch Cancer Institute, and Institute for Translational Epidemiology, Icahn School of Medicine at Mount Sinai, New York, New York, United States of America; 6 Department of Public Health Analysis, School of Community Health and Policy, Morgan State University, Baltimore, Maryland, United States of America; Hospital de Clínicas de Porto Alegre, BRAZIL

## Abstract

**Background:**

Cardiovascular diseases (CVD) are becoming major causes of death in developing countries. Risk scoring systems for CVD are needed to prioritize allocation of limited resources. Most of these risk score algorithms have been based on a long array of risk factors including blood markers of lipids. However, risk scoring systems that solely use office-based data, not including laboratory markers, may be advantageous. In the current analysis, we validated the office-based Framingham risk scoring system in Iran.

**Methods:**

The study used data from the Golestan Cohort in North-East of Iran. The following risk factors were used in the development of the risk scoring method: sex, age, body mass index, systolic blood pressure, hypertension treatment, current smoking, and diabetes. Cardiovascular risk functions for prediction of 10-year risk of fatal CVDs were developed.

**Results:**

A total of 46,674 participants free of CVD at baseline were included. Predictive value of estimated risks was examined. The resulting Area Under the ROC Curve (AUC) was 0.774 (95% CI: 0.762-0.787) in all participants, 0.772 (95% CI: 0.753-0.791) in women, and 0.763 (95% CI: 0.747-0.779) in men. AUC was higher in urban areas (0.790, 95% CI: 0.766-0.815). The predicted and observed risks of fatal CVD were similar in women. However, in men, predicted probabilities were higher than observed.

**Conclusion:**

The AUC in the current study is comparable to results of previous studies while lipid profile was replaced by body mass index to develop an office-based scoring system. This scoring algorithm is capable of discriminating individuals at high risk versus low risk of fatal CVD.

## Introduction

The incidence and overall burden of non-communicable diseases is increasing across the globe [1], and more so in developing countries. In Iran, for example, the share of non-communicable diseases in all-cause mortality increased from 57% in 1990 to 76% in 2010 [2,3]. The major part of this burden is due to ischemic heart disease, stroke, and other vascular diseases, collectively considered as cardiovascular diseases (CVD) [4].

Prevention of CVD is an important step in reducing the burden of these diseases. However, in developing countries, where there are limited resources, prioritizing the allocation of resources based on risk of disease is indeed important. Such risk stratification is possible using a combination of well-established risk factors, such as age, sex, high blood pressure, smoking, dyslipidemia, and diabetes. To provide the best estimate of risk, it is important to use a combination of these risk factors in a model, to avoid over-treatment or under-treatment [5]. Any such model should be clinically useful, methodologically robust, easy to use, clinically addressing relevant risk factors, and resulting in a measurable health gain.

Such risk prediction models have been developed in the past few decades for all CVD combined or their components such as coronary heart disease, stroke, peripheral vascular diseases, and heart failure. These models estimate absolute risk within a certain period of time. The Framingham algorithm has been among the first of this kind developed and re-calibrated [6–9]. A number of other algorithms have been developed and evaluated in different populations [10–22]. The American College of Cardiology / American Heart Association has recently developed sex-specific Pooled Cohort Equations that can be used for non-Hispanic whites and non-Hispanic African Americans, 40–79 years of age [23]. In Iran, two studies have been performed on recalibrating Framingham score, one for estimating 5-year risk of incident CVD and the second on 10-year risk in Tehran. The results have demonstrated that the algorithm is effective in ranking the individuals and quantifying the risk of incident CVD [24,25].

The original and the updated format of Framingham risk scoring and other algorithms adapted from it are based on demographic and life style characteristics as well as laboratory markers of high-density lipoproteins, and either total cholesterol or low density lipoproteins. However, in the setting of primary health care in developing countries, laboratory markers may not always be available. Therefore, algorithms that are based solely on office-based data are preferable. The Framingham algorithm includes an office-based version that is based on age and sex, body mass index (BMI), systolic blood pressure (SBP) stratified by hypertension treatment, current smoking, and diabetes [8]. In the current study, we recalibrated the office-based Framingham algorithm for predicting CVD mortality in the setting of Golestan Cohort in North-East of Iran. If such algorithm is useful and valid, it can be used in the setting of health houses that cover the rural populations with little access to laboratory tests.

## Materials and Methods

### Golestan Cohort Study Design

The details of Golestan Cohort Study have been described elsewhere [26–28]. In brief 50,045 participants aged 40 to 75 years were recruited from January 2004 to June 2008, from those living in Gonbad city and 326 villages in Golestan province, northeast of Iran. Trained interviewers collected data on demographic and life style characteristics, past history, family history, and medication history, using structured questionnaires. Height, weight, and blood pressure were measured.

Nearly all study participants were followed by annual telephone calls. If after seven attempts during a two week period, a participant was not accessible through telephone calls, researchers contacted friends or local health workers. If a death was reported by telephone calls or local health workers or the death registry, a verbal autopsy was completed by a physician and at the same time, all available medical documents were collected within the entire province or the neighboring provinces.

In 35% of cases, the cause of death was determined using verbal autopsy only [27], and for the rest, extensive medical documents were examined in addition to verbal autopsy. Previous studies demonstrated that verbal autopsy was accurate in determining the causes of death, especially for major causes [27]. Using the collected documents, two independent internists determined the cause of death based on ICD-10 codes. If the two were concordant, a diagnosis was made. Otherwise, the final diagnosis was made by a third more senior internist [27].

### Exposures and Outcomes of Interest for the Current Analysis

The exposures of interest in the current analysis included age, sex, body mass index, systolic blood pressure, treatment of hypertension, current smoking, and diabetes. Data on these exposures were collected at cohort study baseline. Blood pressure, height and weight were measured in the health center. Blood pressure was recorded after 5 minutes of rest and in sitting position, twice from each arm with 10-minute intervals, using Richter auscultatory sphygmomanometers. Smoking and receiving anti-hypertensive treatment were documented using self-report. Diabetes was recorded if the study participant reported to be a diabetic, or medical documents or medication history showed he/she was being treated for diabetes.

The outcomes of interest were deaths caused due to CVD, which included ischemic heart disease, cerebrovascular diseases, and all other vascular diseases. Using ICD-10, the following three categories constituted the outcomes of interest:

1. Ischemic Heart Diseases (I20-I25)
2. Cerebrovascular Diseases (I60-I69)
3. Other vascular diseases including:
  - Hypertensive Heart Diseases (I10-I15)
  - Pulmonary Heart Diseases (I26-I28)
  - Other forms of Heart Diseases (I30-I52) including a) cardiac arrest b) heart failure
  - Peripheral Vascular Diseases (I70-I89)

### Statistical Analysis

The purpose of this analysis was to recalibrate the Framingham office-based risk scoring tool for estimating the absolute 10-year risk of CVD deaths and for differentiating the high risk from low risk individuals at clinic or office.

After confirming the assumption of proportionality of hazards, we fitted overall and sex-specific Cox proportional hazards models to correlate the exposures of interest, described above, to CVD mortality during a maximum follow-up of 10 years. The resulting CVD risk functions were used to estimate the sex-specific 10-year absolute risk of CVD mortality for each individual using the formula:

$$\hat{p}=1-S_{0}{\left(t\right)}^{\text{exp}(\sum_{i=0}^{p}\beta_{i}x_{i}-\sum_{i=0}^{p}\beta_{i}{\bar{x}}_{i})}$$;

where S_0_(t) is baseline survival at follow-up time t, *β*_*i*_ is estimated regression coefficient, *X*_*i*_ is naturally log transformed value of the i^th^ risk factor if continuous, ${\overset{-}{X}}_{i}$ is the corresponding mean, and *p* is the number of risk factors. We estimated the 10-year baseline survival using a Kaplan-Meier estimate. The statistical methods were adopted from D’Agostino et al [8].

The covariates used in these models were age, sex, body mass index, systolic blood pressure (SBP), blood pressure treatment, current smoking, and diabetes (self-report or history of medication). The continuous variables were naturally logarithmically transformed to improve calibration and minimize the effect of outliers. Beta coefficients and Hazard Ratios (HRs) were estimated for each one unit increase in naturally logarithmic transformed value of BMI and SBP and for each 0.1 unit increase in naturally logarithmic transformed value of age.

Area under the receiver-operating-characteristics (ROC) curve was used to evaluate the ability of the model to discriminate high-risk from low-risk individuals for being prone to CVD death. The observed versus predicted probabilities in deciles of predicted probability were used to determine the calibration in more detail. Kaplan-Meier estimator was used to obtain observed probabilities. To evaluate the clinical usefulness of the model, we used an array of thresholds for predicted probabilities to estimate the sensitivity and specificity of the model. Thresholds between 2% to 5%, with 0.5% intervals were examined to categorize the predicted risk of CVD death.

All statistical analyses were done using Stata statistical software version 11 (StataCorp, College Station, TX).

### Ethical Considerations

The Golestan Cohort Study was approved by the Institutional Review Boards of the Digestive Disease Research Institute of Tehran University of Medical Sciences in Iran, the US National Cancer Institute, and the World Health Organization International Agency for Research on Cancer. All study participants signed informed consents. The records and identification of participants were anonymized and de-identified prior to statistical analysis.

## Results

Of the 50,045 participants in Golestan cohort, 3,371 participants were excluded due to a history of heart disease or stroke at baseline. The remaining 46,674 participants were followed for a median of 7.1 years (a total of 328,475 person-years of follow-up) and were included in the analyses.

The mean age (SD) of participants at the beginning of the study was 51.6 (8.8) years. A large proportion of the participants were women (57.5%), of Turkmen ethnicity (76.0%), rural dweller (80.8%), married (88.2%), illiterate (69.8%), and currently non-smoker (89.0%). The mean body mass index (SD) was 26.6 (5.4) Kg/m^2^. The mean systolic blood pressure (SD) was 127.0 (23.9) mmHg. A total of 6,802 participants (14.6%) were receiving anti-hypertensive treatment and a total of 2,920 participants (6.3%) suffered from diabetes. The detailed characteristics by sex are presented in Table 1.

**Table 1:** Summary statistics of CVD risk factors in the Golestan Cohort.

	**Women**		**Men**	
Continuous Variables				
	Total Number	Mean (SD)	Total Number	Mean (SD)
Age at enrollment	26,854	51.1 (8.4)	19,820	52.3 (9.3)
Body mass index		27.7 (5.7)		25.1 (4.6)
Systolic blood pressure		128.0 (24.6)		125.5 (22.8)
Categorical variables				
	N positive	% positive	N positive	% positive
Hypertension treatment	5,271	19.6	1,531	7.7
Current Smoking	280	1.1	4,844	24.4
Diabetes	2,013	7.5	907	4.6

A total of 1,438 deaths occurred due to CVD (crude rate = 438/100,000 person years), 653 in women (342/100,000) and 785 in men (570/100,000). The subcategories of causes of death included ischemic heart disease (n = 829), cerebrovascular diseases (n = 483), and other vascular diseases (n = 126).

The adjusted regression coefficients and hazard ratios for determinants of CVD mortality are presented in Table 2. Beta coefficients were positive for all determinants except for natural logarithm of BMI, the coefficient of which was negative in both women and men. However, the negative coefficient was significant only in women and thus, was included in the model. This finding has been reported in previous publications of Golestan Cohort Study as well. We repeated the analyses after excluding the underweight group, and the results did not change.

**Table 2:** Regression coefficients and hazard ratios for CVD mortality.

**Women: (10-year Baseline Survival: S**_**0**_ **(10) = 0.9696914**					
Variable	Beta	P-value	HR	95% CI	
Log of age*	0.4680452	<0.001	1.60	1.52	1.68
Log of BMI	-1.13051	<0.001	0.32	0.22	0.48
Log of SBP if not treated	1.250474	<0.001	3.49	2.25	5.43
Log of SBP if treated	1.395161	<0.001	4.04	2.63	6.20
Smoking	0.835265	<0.001	2.31	1.40	3.80
Diabetes	0.778785	<0.001	2.18	1.78	2.67
**Men: (10-year Baseline Survival: S**_**0**_ **(10) = 0.9504544**					
**Variable**	**Beta**	**P-value**	**HR**	**95% CI**	
Log of age*	0.4294706	<0.001	1.54	1.47	1.61
Log of BMI	-0.06352	0.766	0.94	0.62	1.43
Log of SBP if not treated	1.894138	<0.001	6.65	4.36	10.13
Log of SBP if treated	2.011603	<0.001	7.48	4.95	11.30
Smoking	0.429651	<0.001	1.54	1.30	1.81
Diabetes	0.656457	<0.001	1.93	1.52	2.44

*The Beta coefficients and HRs are estimated for each 0.1 unit increase in naturally logarithmically transformed value of age. For other continuous variables, each one unit increase has been used.

**Fig 1:**
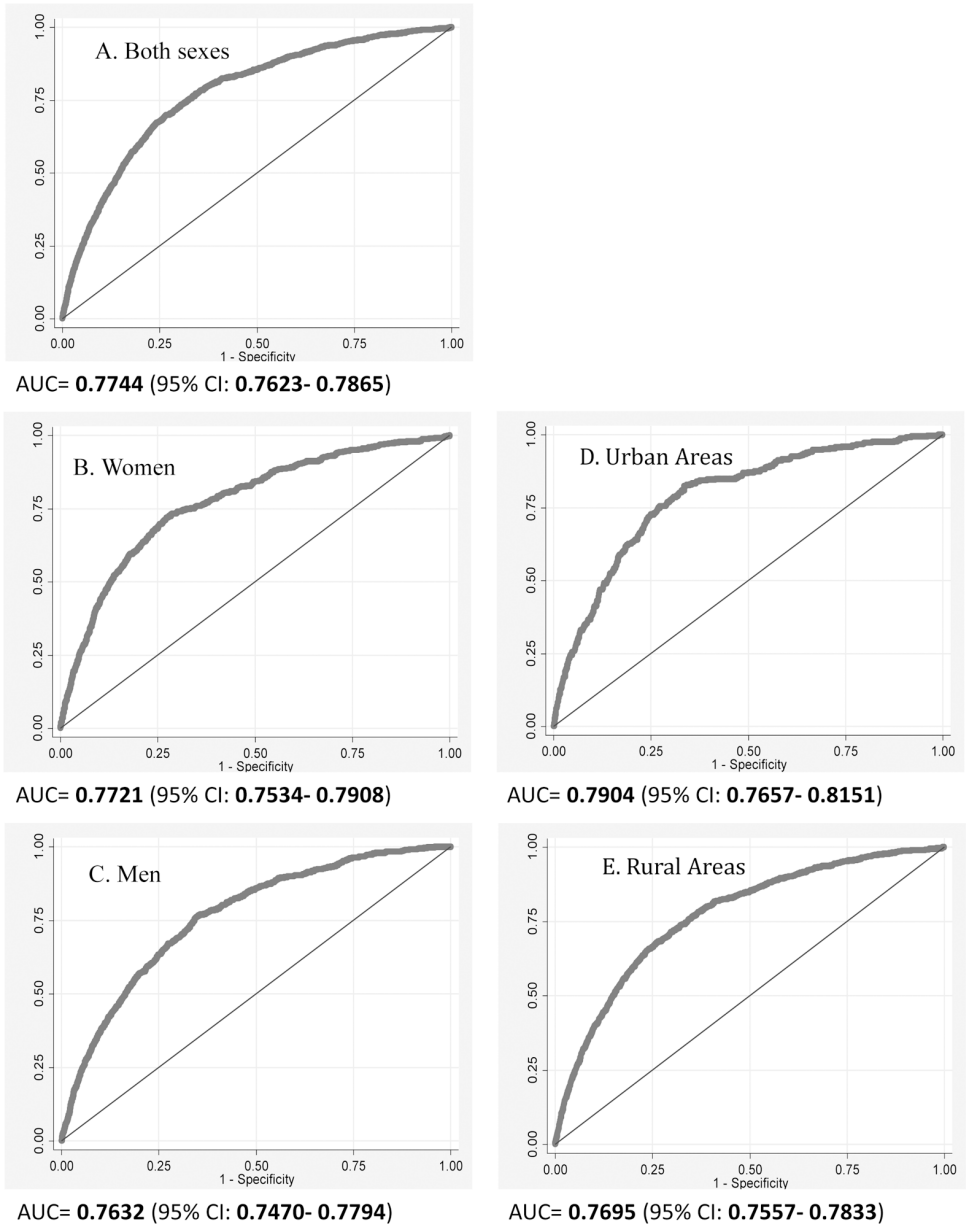
ROC curves of observed 10-year risk versus predicted risk of fatal CVD.

The overall and sex-specific CVD death risk functions were estimated. The area under ROC Curve was 0.774 (95% CI: 0.762–0.787) in all participants, 0.772 (95% CI: 0.753–0.791) in women, and 0.763 (95% CI: 0.747–0.779) in men (Fig 1). The areas under the curve were close for urban areas with AUC of 0.790 (95% CI: 0.766–0.815), and rural areas with AUC (95% CI) of 0.770 (95% CI: 0.756–0.783).

**Fig 2:**
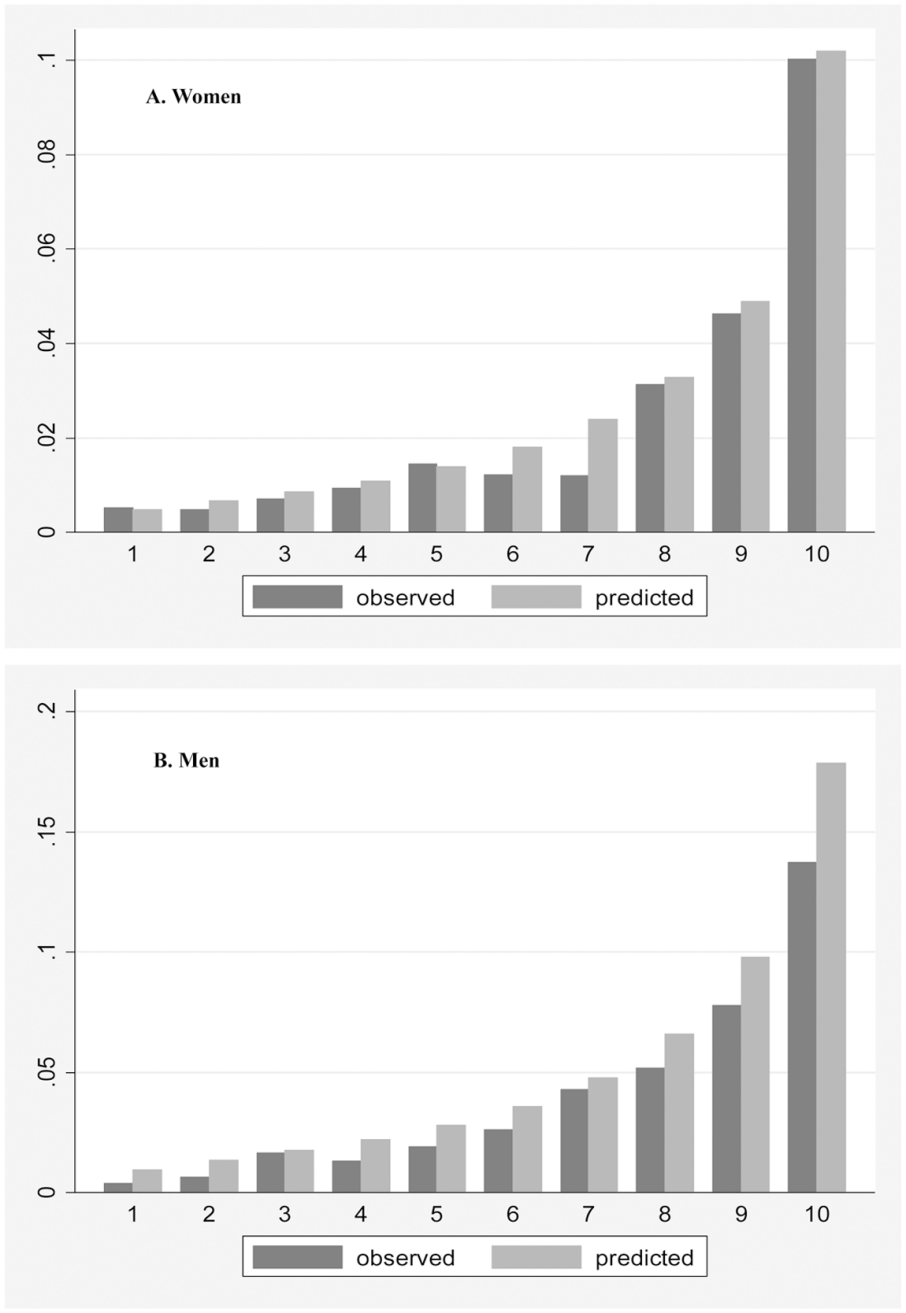
Ten-year predicted versus observed risk of CVD deaths in the Golestan Cohort Study participants. The X axis shows the deciles of predicted risks. The Y axis shows the mean risk.

Fig 2 demonstrates the observed probability of CVD deaths versus the predicted probabilities separately in women and men. The observed and predicted probabilities were similar for women. However, for men, the predicted probabilities were modestly higher than the observed ones, particularly in the categories that had a predicted high risk.

Table 3 demonstrates the clinical performance of the model using 7 thresholds for predicted 10-year risk of fatal CVD: 2%, 2.5%, 3%, 3.5%, 4%, 4.5%, and 5%. In women, the threshold of 3% performs best, while in men the best threshold is 4.5%

**Table 3:** The sensitivity and specificity of the model in predicting 10-year risk of CVD deaths in the Golestan Cohort Study participants according to different levels of predicted 10-year risk chose as threshold between low risk and high risk individuals.

	**Threshold of 2%**		**Threshold of 2.5%**		**Threshold of 3%**		**Threshold of 3.5%**	
	Sensitivity (95% CI)	Specificity (95% CI)	Sensitivity (95% CI)	Specificity (95% CI)	Sensitivity (95% CI)	Specificity (95% CI)	Sensitivity (95% CI)	Specificity (95% CI)
Women	78.8 (75.5–81.9)	59.8 (59.2–60.4)	74.7 (71.2–78.0)	67.7 (67.1–68.3)	70.7 (67.0–74.2)	73.5 (73.0–74.0)	64.0 (60.1–67.6)	78.1 (77.6–78.6)
Men	93.0 (91.0–94.7)	31.5 (30.8–32.2)	89.8 (87.5–91.8)	41.7 (41.0–42.4)	86.0 (83.3–88.3)	49.4 (48.7–50.2)	82.3 (79.4–84.9)	55.6 (54.9–56.3)
All	86.6 (84.7–88.3)	47.9 (47.4–48.3)	82.9 (80.9–84.9)	56.8 (56.3–57.2)	79.0 (76.8–81.1)	63.4 (62.9–63.8)	74.0 (71.6–76.2)	68.7 (68.2–69.1)
	**Threshold of 4%**		**Threshold of 4.5%**		**Threshold of 5%**			
	Sensitivity (95% CI)	Specificity (95% CI)	Sensitivity (95% CI)	Specificity (95% CI)	Sensitivity (95% CI)	Specificity (95% CI)	-	-
Women	59.5 (55.6–63.3)	81.8 (81.3–82.2)	54.4 (50.5–58.3)	84.5 (84.1–85.0)	50.8 (46.9–54.7)	86.7 (86.3–87.1)	-	-
Men	78.6 (75.5–81.4)	60.6 (59.9–61.3)	76.5 (73.4–79.5)	64.8 (64.2–65.5)	71.2 (67.9–74.3)	68.3 (67.7–69.0)	-	-
All	69.9 (67.5–72.3)	72.9 (72.4–73.3)	66.5 (64.0–68.9)	76.3 (75.9–76.6)	61.9 (59.3–64.4)	79.0 (78.6–79.4)	-	-

## Discussion

The aim of the present study was to calibrate an office-based risk scoring system for prediction of 10-year risk of fatal CVD in Iran. The performance of the office-based model is comparable to previously studied lab-based models, with an AUC equal to 0.774 (95% CI: 0.762–0.787) in all participants, 0.772 (95% CI: 0.753–0.791) in women, and 0.763 (95% CI: 0.747–0.779) in men. The predicted and observed 10-year risks of fatal CVD were equivalent in women. However, in men, predicted probabilities were higher than observed.

Whereas previous studies have presented risk scores for CVD, our study is the first large cohort study in the Middle-East with over 50,000 individuals that calibrated the risk scoring system. Furthermore, our study had three distinct characteristics: 1) CVDs were the outcome of interest; 2) mortality was the failure event; and 3) scoring was done using office-based CVD risk factors.

Previous original studies have been mostly done on coronary heart disease as the failure event [7,9,10,13,17], but recent models are based on risk of all CVD combined [8,14,15,19,20,23,25]. This strategy is a reasonable one, as the three main components of CVD (coronary heart diseases, cerebrovascular diseases, and other vascular diseases) are all important causes of burden and share the same set of risk factors. Calculating total CVD risk is also preferred for treatment decisions.

In the current study, mortality has been chosen as the failure event. While both non-fatal and fatal CVD impose substantial medical and economic burden, we used only fatal outcomes. This was in part because, in Golestan Cohort Study, CVD outcomes were recorded much more precisely than non-fatal outcomes. Moreover, mortality results from this cohort can be more easily used and generalized to the country, as mortality by cause is more rigorously reported by the national death registry in Iran. The prominent example of another study that used fatal CVD outcomes, with similar justifications as presented above, is the SCORE project, which was conducted across Europe [11].

The third and the most important characteristic of the present model is the usage of office-based risk factors. In the setting of primary or even secondary care in developing countries, the lab markers of lipids may not be available. Despite excluding serum lipid results from the predictors, our model performed nearly as well as those in other studies that used lipids [11]. The AUC of 0.774 in our study is comparable to the results of SCORE project, in which the reported AUC ranged from 0.67 to 0.82 in different countries in Europe with various levels of CVD risk [11].

In our study, except for BMI, all conventional CVD risk factors were associated with an increased risk of CVD. We strongly believe our findings regarding BMI are correct. Both height and weight were accurately measured at study baseline. The association has been shown in previous studies in Golestan too [29]. Some other cohorts in Asia have also shown relatively weak associations with higher BMI. For example, a large cohort study in China showed a weak association between BMI mortality [30]. This may indicate that the association of BMI with all-cause and CVD mortality may depend on more distal causal factors, i.e., those that lead to higher BMI.

It is not possible to choose a single ideal threshold for 10 year risk of fatal CVD to discriminate high risk from low risk individuals, and optimal thresholds are different by sex and age [31]. In Framingham study, where CVD incidence was the main outcome, thresholds of 10% and 20% were used to discriminate individuals at high risk of incident coronary heart disease from low risk peers, and other studies have followed the rule [8]. In the SCORE project, where CVD death was the main outcome of interest, thresholds of 2% and 5% were chosen to discriminate low- and high-risk individuals. In the current study, a threshold of 3% for women and 4.5% for men performed best. This is consistent with the results of SCORE project, in which the main outcome was similar to ours. However, the thresholds used here, were mostly based on sensitivity and specificity of the model. Decisions on optimal thresholds for action need to consider local economic factors and availability of resources, which is beyond the scope of this paper.

Our study has several points of strength. Data came from a large prospective study with over 99% successful follow-up rate and precise recording of cause-specific deaths using a robustly designed verbal autopsy and detailed collection of existing medical records. Office-based risk factors were measured according to standard methods with intensive training of the staff. Also, the setting of the study is a representative sample consisting of both urban and rural areas.

Our study is prone to a number of limitations. Our estimated risk functions were based on single measurements rather than ‘usual’ levels, which may result in regression dilution bias of our results. There may not be exact concordance between reported use of anti-hypertensive treatments and actual use of these drugs, particularly in rural areas, which may again lead to dilution of the results. Despite these limitations, the risk factors were highly predictive of CVD mortality in this cohort. Furthermore, these results reflected the clinical settings more realistically, where patients may have similar reporting problems.

Our study has several implications for primary and secondary health care in Iran. The rural population of Iran is extensively covered by a primary healthcare system, which is staffed by local health workers called the Behvarz. With the sharp increase in chronic diseases, the functions of this system need to shift toward prevention of chronic diseases, and in particular CVD. Using the risk scoring algorithms makes screening and follow-up of high risk individuals more cost-effective, as differentiating high risk from low risk individuals makes it possible for health care providers to decide on intensity of lifestyle interventions and pharmacologic treatment and intervals of follow-up. The actual function of the scoring algorithm can be evaluated through trials. In fact, clinical trials of Polypills are ongoing in Iran [32–34]. If these trials show success in the use of Polypills, risk scoring systems can prioritize their use in individuals with highest risk. Predictions based on this risk scoring system can help maximize the cost-effectiveness of treatment plans by preventing over- or under-treatment. There remain trials at national scale to further optimize the performance of risk scoring algorithms.

## Conclusions

We were able to validate a risk scoring system for CVD using data from the largest cohort study in the Middle East. Results could be used for prioritizing prevention efforts in the Iranian health system and other developing countries. These findings should guide the identification of individuals at higher risk of developing cardiovascular diseases, who should be the focus of proven non-drug and drug interventions to prevent cardiovascular diseases.

## Supporting Information

S1 Dataset The underlying data of the present study. The excel sheet contains: age, sex, hypertension treatment, current smoking, residence, systolic blood pressure, body mass index, diabetes, cardiovascular death, and follow-up time.
(XLSX)
